# Assessment of the perceived burden associated with Malignant Melanoma with Pictorial Representation of Illness and Self Measure (PRISM) and Melanoma Concerns Questionnaire (MCQ-28)

**DOI:** 10.1007/s00520-021-06760-2

**Published:** 2022-01-15

**Authors:** Alessandro Borghi, Maria Elena Flacco, Alberto Monti, Lucrezia Pacetti, Michela Tabanelli, Monica Corazza

**Affiliations:** 1 Section of Dermatology and Infectious Diseases, Department of Medical Sciences, University of Ferrara, Via L. Ariosto 35, 44121 Ferrara, Italy; 2 Section of Public Health, Department of Medical Sciences, University of Ferrara, Ferrara, Italy; 3 Dermatology Unit AUSL della Romagna, Ravenna, Italy

**Keywords:** MCQ28, Melanoma, Melanoma-related burden, PRISM, Quality of life, Suffering

## Abstract

**Purpose:**

The impact of malignant melanoma (MM) on patients’ psychophysical well-being has been poorly addressed. We aimed to assess the perceived burden in patients with a diagnosis of MM, using two different tools, one generic and one specific for MM, such as Pictorial Representation of Illness and Self Measure (PRISM) and Melanoma Concerns Questionnaire (MCQ-28), respectively. The correlation between PRISM and MCQ-28 subscales and the relevance of disease and patient-related variables were also investigated.

**Methods:**

This single-centre, cross-sectional study included all adult consecutive MM patients who attended our Dermatology Unit from December 2020 to June 2021. Demographics and disease-related data were recorded. PRISM and MCQ-28 were administered.

**Results:**

One hundred and seventy-one patients were included (mean age: 59.5 ±14.9 years.; 48.0% males). Median time from MM diagnosis to inclusion was 36 months. Nearly 80% of the patients had in situ or stage I MM. Overall, 22.2% of the patients reported a PRISM score <100mm and similar percentages provided scores indicating impaired quality of life, as assessed with MCQ-28 subscales. A weak, albeit significant, correlation was found between PRISM scores and ACP, CON and SOC2 subscales. The most relevant association found was that between lower PRISM scores and higher-stage MM.

**Conclusions:**

In the study population, mostly affected with superficial MM, their perception of the burden associated with MM did not appear either particularly dramatic or disabling. PRISM seems a reliable system for capturing and quantifying the domains correlated with the emotive dimension of MM, especially MM-related concerns and willingness to face life

**Supplementary Information:**

The online version contains supplementary material available at 10.1007/s00520-021-06760-2.

## Introduction

Malignant melanoma (MM) is the most aggressive skin cancer and is responsible for about 75% of deaths from skin tumors [1]. Its incidence has considerably risen worldwide during the recent decades, with considerable differences between countries [2–5]. In spite of this, a relatively stable mortality trend has been recorded [6]. Both an increasingly earlier diagnosis and the availability of continuously evolving treatments for more advanced stages account for this observation. As a consequence, about 80% of patients survive MM [6]. MM survivors have a risk of disease progression and/or recurrence as well as of developing second primary MM [7]. Moreover, a long-term and quite frequent clinical, laboratory and instrumental surveillance is recommended, even though a uniform consensus and evidence-based follow-up regimen have not been established [8]. Patients’ lifestyles and behaviours are conditioned and limited, especially regarding ultraviolet exposure, in both occupational and recreational situations. Surgical sequelae as well as the toxic effects of chemotherapy, the latter in advanced stages of the disease, may be health concerns for MM survivors. Anxiety about an increased risk of MM development among relatives can further affect their health-related quality of life. Therefore, for such patients, MM can be considered a chronic, hugely distressing disease [9].

To date, the impact of MM on patients’ quality of life as well as on their physical and psychic well-being has not been deeply addressed [10, 11]. On the one hand, this is quite surprising, given the growing sensitivity towards the psychosocial issues of patients, especially oncological patients [12]. On the other hand, to assess a disease burden and its impact on patients’ health may be extremely difficult. In fact, it is recognized that the extent of suffering and quality of life deterioration associated with illness is determined by several factors, which include both the direct effects of the disease itself and the perception that a subject has of his/her own state. In other words, the intensity of suffering has multidimensional and heterogeneous determinants. In keeping with this, reliable and sensitive tools should at the same time capture and measure psychological, emotional, physical and social issues. The more a tool is able to identify the specific, critical issues of a certain disease, the more it fulfills its function of duly measuring the disease-related burden and suffering. A few questionnaires have been specifically designed for use with MM patients, namely the Malignant Melanoma Module [13], the FACT-Melanoma (FACT-M) [14], the EORTC Melanoma Module (QLQ-MEL38) [15] and its revised version, the Melanoma Concerns Questionnaire (MCQ-28) [9]. The questionnaires developed by the EORTC appear particularly suitable for the assessment and quantification of MM-related issues, which are grouped by domains [9, 15, 16].

In clinical practice, unlike in trials, there is the need for rapid tools, easily understood by subjects of any cultural and social level. Our group has recently tested the use of Pictorial Representation of Illness and Self Measure (PRISM) for assessing the burden of suffering from inflammatory skin disorders, in particular of the genital area [17, 18]. It is a visual, non-verbal instrument aimed at quantitatively assessing the burden of illness-related suffering [19–21]. It requires simple instructions and little time to complete and is particularly suitable for facilitating doctor-patient communication on ‘difficult-to-verbalize’ issues [19]. PRISM has proved to be valid and reliable in various conditions, including cancer [22].

The present study had two main objectives: first, to assess to what extent MM impacts survivors’ well-being and quality of life, in relation to several variables, both disease and patient-related. Second, we were interested in evaluating the capability of PRISM to assess them, as compared with a questionnaire specifically designed to assess the quality of life in melanoma patients, such as the MCQ-28.

## Materials and methods

### Study design and objectives

The present study was set up as a single-centre, cross-sectional study. The main objectives of the study were to investigate the following: (i) the perceived suffering and quality of life impairment in patients with a previous diagnosis of MM assessed by PRISM and MCQ-28; (ii) the degree of correlation between PRISM and each MCQ-28 subscale in measuring illness burden; (iii) the impact of selected clinical and demographic characteristics on the degree of suffering and quality of life impairment.

This was a spontaneous survey, with no funding from external sources. The study was approved by the University-Hospital of Ferrara institutional review board (EM107-2021_644/2020/Oss/AOUFe – EM1). Patients provided their written informed consent.

### Study patients

All adult (≥18 years) consecutive patients with a histological diagnosis of MM who attended the Dermatology Unit of the University of Ferrara, Italy, between December 2020 and June 2021 were considered for eligibility. To be included, patients had to be either new patients or follow-up patients. There were no exclusion criteria with regard to stage, time from diagnosis, clinical course, multiple MM and current or previous medical treatments. Previous or concomitant diseases were not exclusion criteria. For patients with more than one melanoma, the most “severe”, i.e. the one with a higher stage, was taken as the relevant lesion for the objectives of this study. Refusal or inability to answer the two questionnaires, namely PRISM and/or MCQ-28, was the sole exclusion criterion.

### Data collection

The following data were recorded by interview through a verbally administered questionnaire: (1) age at inclusion; (2) marital status (single/never married; married/domestic partnership; widowed; divorced/separated); (3) educational level (primary school; intermediate school; high school; university degree); (4) employment (full-/part-time employed; unemployed/looking for work; student; retired; homemaker); (5) major previous or concomitant diseases; (6) time between MM histological diagnosis and study inclusion; (7) stage of MM, according to the eighth edition of American Joint Commission on Cancer melanoma staging system [23]; (8) MM site (trunk; upper or lower limbs, others: face, scalp, hands, feet or genitals); (9) multiple or single lesions; (10) MM therapy, including both medical treatments, defined as documented courses with any pharmacological active administered for MM and radiation therapy.

PRISM was administered to all participants, while MCQ-28 [9], which was developed at the University of Sydney and translated into Italian for this study, was kindly provided by the authors in January 2021. For this reason, MCQ-28 was administered to the patients included from that time and not to those included in the previous weeks after the study began. Both questionnaires were administered by the same investigator at study recruitment. In the present study, we used the original version of PRISM [19] and the Italian translation of MCQ-28.

#### PRISM

The PRISM test was performed by showing the patient an A4-sized (210 × 297mm) white sheet of paper with a printed yellow disc 6 cm in diameter at the bottom right-hand corner (Supplementary Figure). The patient was told that the white sheet of paper represents the “patient’s life at the moment” and the yellow the patient’s “self”. In the next step, a cardboard red disc, 4 cm in diameter, which represents the patient’s disease, namely the melanoma, was handed to the patient. We explained to the patient that by “melanoma” we meant all aspects related to the disease, including fear, anxiety, worry, the need to undergo periodic checkups and visits, functional limitations and/or aesthetical impairments by surgical intervention/s and side effects of treatments. The patient was then asked to place this red disc onto the sheet after being asked: ‘Where would you locate your illness (the red disc) in your life (the sheet) at this moment?’ The distance between the two disc centres, called the Self-Illness Separation (SIS), which ranges from 0 to 273 mm, reflects the patient’s burden of suffering. Lower SIS scores indicate greater suffering while higher scores correspond to a lower impact of the disease.

#### Melanoma Concerns Questionnaire (MCQ-28)

The MCQ-28 consists of a total of 28 items, of which 23 items are grouped in four subscales and 5, grouped in two subscales, are scored individually (Supplementary Table). The subscales, which span several psycho-oncological domains deemed important to melanoma patients, include the following: (i) disease prognosis and acceptance (abbreviated ACP, 6 items, each scored on a 4-point scale, raw score range 0–18), (ii) treatment concerns/future disease risk (CON, 8 items, each scored on a 4-point scale, raw score range 0–24), (iii) care delivery/communication (CARE, 3 items, each scored on a 3-point scale, raw score range 0–6), (iv) supportive care (SUP, 6 items, each scored on a 3-point scale, raw score range 0–12). Melanoma surgery site includes 3 individual items (SURG1, SURG2, SURG3, each scored on a 4-point scale, raw score range 0–3) and social circumstances include 2 individual items (SOC1, SOC2, each scored on a 4-point scale, raw score range 0–3). The patients completed the questionnaire independently, with a guarantee of support from the investigators for any request for clarification in respect of issues of the questionnaires that may be unclear. For ACP, CARE, SUP and SOC subscales, higher scores correspond to a higher quality of life. For CON and SURG subscales, a higher score indicates a lower quality of life.

### Statistical analysis

Initial descriptive statistics were used to analyze self-reported quality of life, assessed through the PRISM score (expressed in mm) and through each subscale of the MCQ-28 (namely ACP, CON, CARE, SUP, SOC1-2 and SURG1-3). Then, the correlation between the PRISM score and (1) ACP; (2) CON; (3) CARE; (4) SUP; (5) SOC1; and (6) SOC2 subscales was evaluated computing the corresponding Spearman correlation coefficients (Spearman’s rho), and fitting six univariate regressions.

Second, the potential association of (1) PRISM score; (2) disease prognosis and acceptance (ACP) subscale; (3) treatment concerns/future disease risk (CON) subscale; (4) care delivery/communication (CARE) subscale; (5) supportive subcare (SUP); and (6) social circumstances (SOC1-2) subscales with selected demographic and clinical parameters was evaluated fitting seven multiple regression models. All recorded covariates, which had been tested previously for multicollinearity, were included a priori and potential transformation, interaction and/or quadratic/cubic terms were investigated. In all models, cancer stage and educational level were treated ordinally, including the different melanoma stages and levels of education as dummy variables. Additionally, due to the relatively small number of patients with MM stage ≥ II, stages II, III and IV were grouped and analyzed together. No analysis was performed for melanoma surgery site (SURG1-2) scales, due to the high number of missing values in these items. The validity of final regression models was assessed as follows. The assumption of constant error variance was checked graphically, plotting Pearson’s residuals vs. fitted values, and formally, using the Cook-Weisberg test for heteroskedasticity. High-leverage observations were identified by computing Pearson’s, standardized and studentized residuals, Cook’s D influence and the hat diagonal matrix.

Statistical significance was defined as a two-sided *p*-value <0.05 for all analyses, which were carried out using Stata version 13.1 (Stata Corp., College Station, TX, 2014).

## Results

### Overall characteristics

One hundred and seventy-two patients were eligible for the study. One refused to give consent so data from 171 subjects were analyzed. Patients were evenly distributed by sex, with an overall mean age of 59.5 years (SD = 14.9). Median time from MM diagnosis to study inclusion was 36 months (interquartile range, IQR: 50.0). Nearly 80% of the patients had a superficial melanoma (in situ or stage I), most commonly localized on the trunk (50.3%) (Table 1).

**Table 1:** Selected demographic and clinical characteristics of the sample

	Overall sample
Variables	(*n*=171)
Mean age in years (SD)	59.5 (14.9)
Male gender, %	48.0
Marital status, %	
- Single	16.9
- Married/cohabiting	66.7
- Divorced/separated	9.4
- Widowed	7.0
Educational level, %	
- Primary school	14.1
- Secondary school	25.9
- High school	34.1
- University/higher	25.9
Employment, %	
- Full-/part-time employed	45.3
- Retired	45.3
- Other (student, household)	5.3
- Unemployed/looking for work	4.1
Comorbidities, %	
- None	56.1
- Cardiovascular diseases	17.5
- IGT/Diabetes	12.3
- Neurological diseases	5.3
- Multiple diseases	8.8
Melanoma site, %	
- Trunk	50.3
- Upper limbs	18.7
- Lower limbs	22.2
- Other *	8.8
Cancer stage at diagnosis, %	
- In situ	18.1
- IA-IB	60.8
- II-III-IV	21.1
Presence of multiple lesions, %	5.3
Time from surgical resection in months, %	
- <24	27.5
- 24–35.9	21.6
- 36–71.9	25.7
- ≥72	25.2
Median time in months (IQR)	36.0 (50.0)

*SD* standard deviation; *IGT* impaired glucose tolerance; *IQR* interquartile range

*Face, scalp, hands, feet or genitals

### PRISM and MCQ-28 scores

Table 2 shows the frequency distributions of the data from the PRISM and MCQ-28 questionnaires. All the included subjects responded to PRISM, while MCQ-28 could be administered to 140 patients, since it was not available in the first 2 months of inclusion. Additionally, the answers to some items of MCQ-28 investigating social (SOC1 and SOC2) and surgical (SURG1, SURG2 and SURG3) issues were available only for 73 (SOC1), 117 (SOC2) and 13 (SURG1-3) patients, respectively. The lack of response to the aforementioned items was due to the fact that they were not relevant for all patients. In particular, SOC1 was not relevant for non-workers, SOC2 for subjects without intimate relationships at the time of completing the questionnaire and SURG1-3 for patients whose MM had been removed for more than 12 months. Overall, 22.2% of the patients reported a PRISM score <100mm; a total of 20.7%, 16.6% and 27.2% of the subjects showed values <9 for the ACP subscale, ≤2 (CARE subscale) and <6 (SUP subscale), respectively. Scores ≥9 (CONC subscale) were reported by 25% of the participants. Values of SOC items ≤1 were recorded in 28.8% (SOC1) and 12.8% (SOC2) of the participants, respectively.

**Table 2:** Results of the Pictorial Representation of Illness and Self Measure (PRISM) score and the Melanoma Concerns Questionnaire (MCQ-28) in the overall sample

*PRISM score:*	
Median score in mm (IQR)	200 (120–270)
- Min–max values	0–270
Distribution of patients by score category, %	
- <100mm	22.2
- 101–149mm	10.5
- 150–199mm	15.8
- 200–249mm	16.4
- ≥ 250mm	35.1
*MCQ-28 subscales*:	
1. Disease prognosis and acceptance (ACP)	(*n*=140)
- Min–max values	0–18
- Median score (IQR)	12.0 (9–14)
Distribution of patients by score category, %	
- <9	20.7
- 9-11	25.0
- ≥ 12	54.3
2. Treatment concerns/future disease risk (CON)	(*n*=140)
- Min–max values	0–16
- Median score (IQR)	5.0 (4–8.5)
Distribution of patients by score category, %	
- <6	50.7
- 6–8	24.3
- ≥ 9	25.0
3. Care delivery/communication (CARE)	(*n*=139)
- Min–max values	1–6
- Median score (IQR)	4.0 (3–5)
Distribution of patients by score category, %	
- 1–2	16.6
- 3–4	51.0
- 5–6	32.4
4. Supportive care (SUP)	(*n*=140)
- Min–max values	1–12
- Median score (IQR)	7.5 (5–10)
Distribution of patients by score category, %	
- <6	27.2
- 6–8	37.1
- ≥ 9	35.7
5. Social circumstances	
- SOC1	(*n*=73)
- Min–max values	0–3
Distribution of patients by score category, %	
- 0–1	28.8
- 2	23.3
- 3	47.9
- SOC2	(*n*=117)
- Min–max values	0–3
Distribution of patients by score category, %	
- 0–1	12.8
- 2	40.2
- 3	47.0
6. Melanoma surgery site *	
- SURG1	(*n*=13)
- % subjects with score = 1	7.7 (*n*=1)
- % subjects with score = 0	92.3 (*n*=12)
- SURG2	(*n*=13)
- % subjects with score = 1	15.4 (*n*=2)
- % subjects with score = 0	84.6 (*n*=11)

*IQR* interquartile range.

*Questionnaire submitted only to those patients who underwent melanoma resection ≤12 months before

For subscales CON and SURG, a higher score indicates a lower quality of life; for the remaining subscales of the MQC-28 questionnaire, and for PRISM scale, a lower score indicates a lower quality of life

**Fig. 1.**
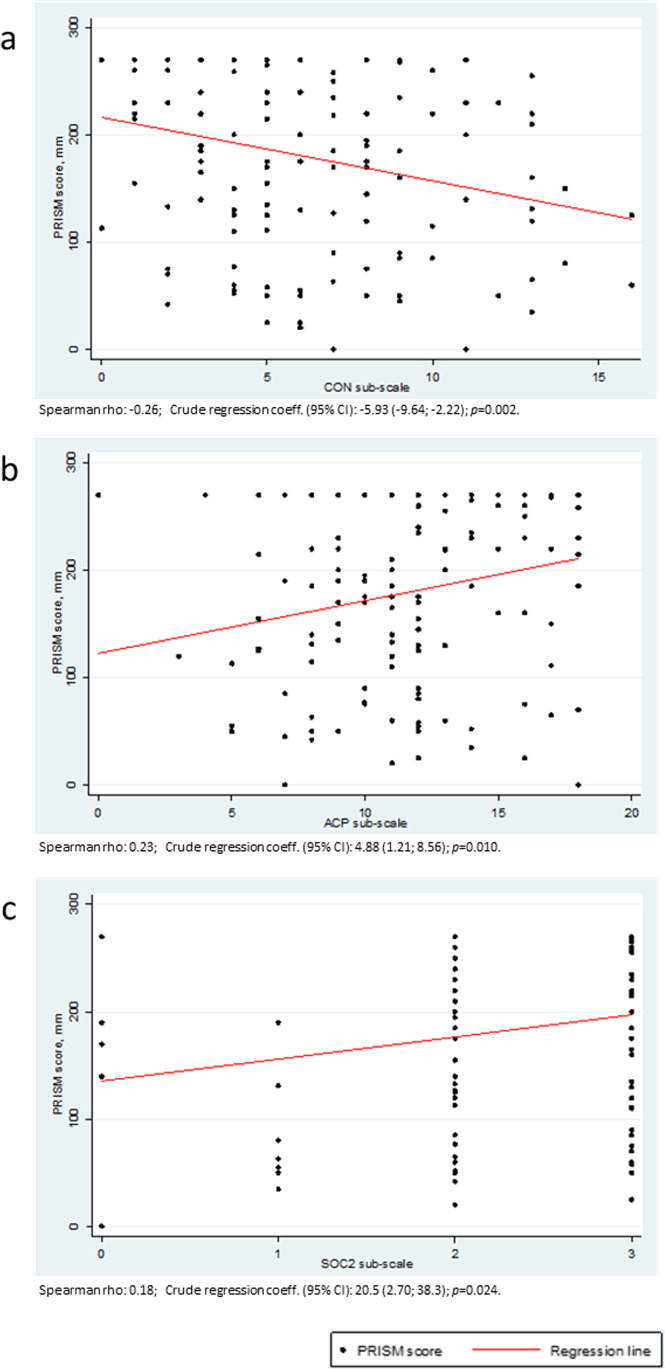
Scatter plot (and regression line) of the Pictorial Representation of Illness and Self Measure (PRISM) score variation (expressed in mm) versus **a** the treatment concerns/future disease risk (CON) subscale variation, **b** the disease prognosis and acceptance (ACP) subscale variation, **c** the social circumstances (SOC2) subscale variation

**Fig. 2.**
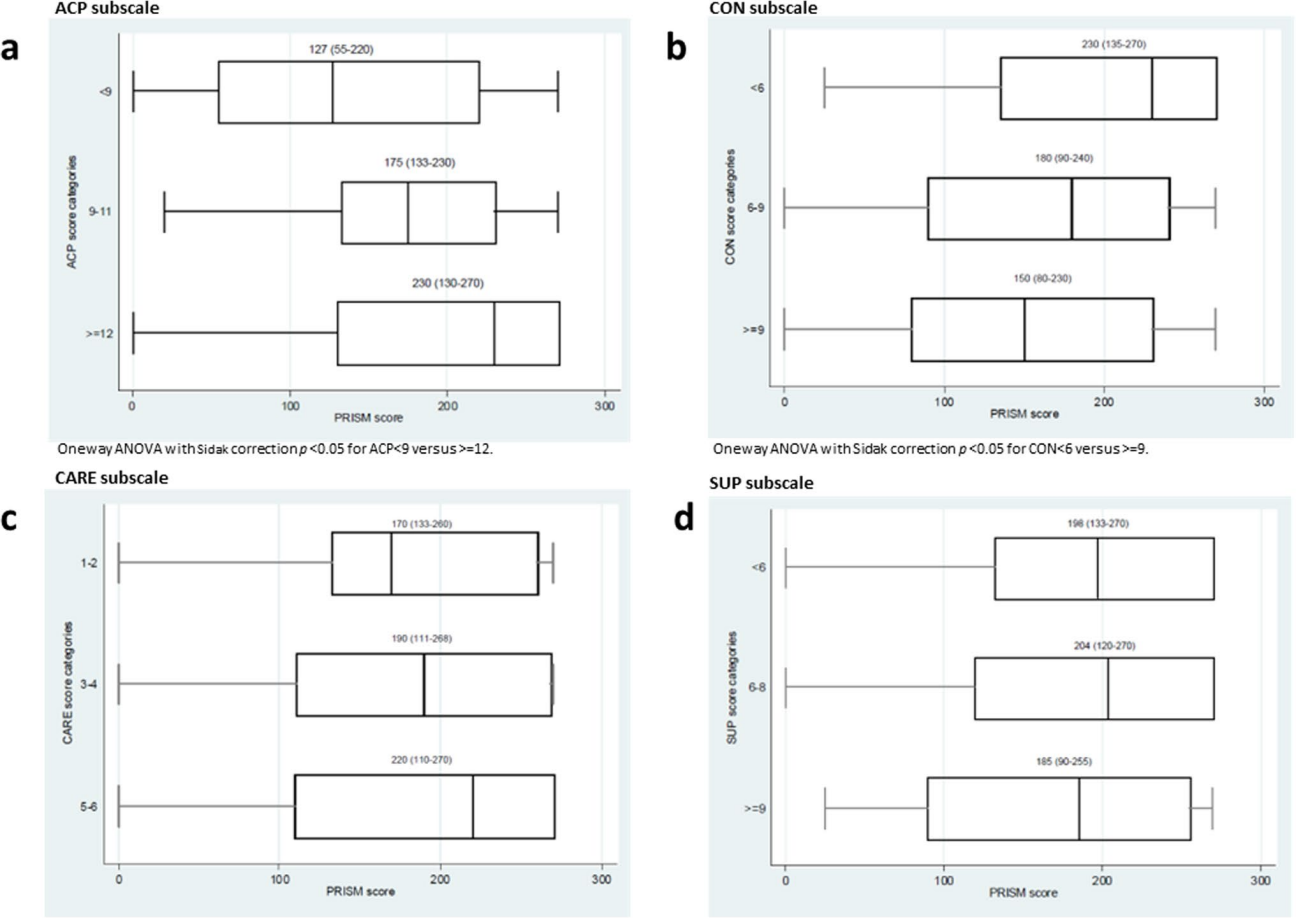
PRISM score across categories of each MCQ-28 subscale. Numbers are median and (IQR) values of PRISM scale, by each category

Univariate analysis showed a weak (although significant), correlation between PRISM scores and some MCQ-28 subscales, namely ACP, CON and SOC2 (Table 3 and Fig. 1). The median values of PRISM across categories of ACP, CON, CARE and SUP subscales have been shown in Fig. 2. PRISM score was significantly higher among the subjects with ACP values ≥12, as compared to those with ACP<9, and significantly lower among those subjects reporting CON values ≥9, versus the lowest score class.

**Table 3.** Correlation coefficients (Spearman’s rho), and crude regression coefficients between Pictorial Representation of Illness and Self Measure (PRISM) score and five of the six subscales of the Melanoma Concern Questionnaire (MCQ-28)

MCQ-28 questionnaire subscales	Spearman’s rho	Crude coeff. (95% CI)
1. Disease prognosis and acceptance (ACP)	**0.23**	**4.88 (1.21; 8.56)**
2. Treatment concerns/future disease risk (CON)	**−0.26**	**−5.93 (−9.64; −2.22)**
3. Care delivery/communication (CARE)	0.06	1.79 (−8.32; 11.9)
4. Supportive care (SUP)	−0.06	−2.04 (−7.17; 3.09)
5. Social circumstances (SOC):		
- SOC1	−0.002	−3.07 (−18.6; 12.5)
- SOC2	**0.18**	**20.5 (2.70; 38.3)**
6. Melanoma surgery site (SURG) *		
- SURG1	**−0.08**	**−0.00 (−0.02; 0.02)**
- SURG2	**0.17**	**0.01 (−0.02; 0.03)**
- SURG3	**0.08**	**0.01 (−0.03; 0.05)**

*Coeff.* coefficient; *CI* confidence interval

Significant results are reported in bold

*Data available only for the 13 patients who underwent melanoma resection ≤12 months before

### Association between PRISM and MCQ-28 scores and selected demographic and clinical variables

After adjusting for selected disease-specific and demographic characteristics, the only variable significantly associated with a lower self-perceived illness (assessed by the PRISM scale) was a lower-stage MM at diagnosis: as compared to patients with in situ lesions, those with a II+ stage had significantly lower PRISM scores (adjusted coefficient: −44.8; 95% confidence interval, CI: −87.2; −2.43, *p*=0.038). No other variables significantly affected PRISM or MCQ-28 scores, with the exception of educational level (patients with higher degrees versus primary school diploma showed significantly lower SUP scores—all *p*<0.05) and higher-stage MM, which was associated with a small, but significant, increase in the self-reported SOC2 scores (*p*<0.05 for subjects with IA-IB stages versus those with a cancer in situ) (Table 4).

**Table 4:** Multiple linear regression: relationship between PRISM score, ACP, CON, CARE, SUP, SOC1 and SOC2 and selected demographic and clinical variables

	PRISM score		ACP scale		CON scale		CARE scale		SUP scale		SOC1 scale		SOC2 scale	
*Covariates*	Adjusted coeff. (95% CI)	*p*	Adjusted coeff. (95% CI)	*p*	Adjusted coeff. (95% CI)	*p*	Adjusted coeff. (95% CI)	*p*	Adjusted coeff. (95% CI)	*p*	Adjusted coeff. (95% CI)	*p*	Adjusted coeff. (95% CI)	*p*
Age, 10-year increase	8.11 (−5.33; 21.6)	0.2	−0.57 (−1.23; 0.09)	0.09	−0.21 (−0.88; 0.47)	0.4	−0.10 (−0.35; 0.16)	0.5	**−0.50 (−0.98; 0.02)**	**0.040**	−0.25 (−0.60; 0.10)	0.16	−0.04 (−0.21;0.12)	0.8
Male gender	16.3 (−11.6; 44.3)	0.3	0.49 (−0.89; 1.86)	0.5	−0.57 (−1.97; 0.83)	0.5	0.34 (−0.19; 0.87)	0.2	−0.81 (−1.80; 0.18)	0.11	0.37 (−0.29; 1.04)	0.3	0.04 (−0.29; 0.37)	0.6
Married Yes vs no	−6.11 (−34.9; 22.7)	0.7	−0.64 (−2.08; 0.80)	0.4	0.18 (−1.28; 1.65)	0.8	0.29 (−0.27; 0.84)	0.3	0.67 (−0.37; 1.70)	0.2	−0.31 (−0.97; 0.36)	0.4	−0.05 (−0.43;0.32)	0.8
Educational level:														
•Primary school	0 (ref. cat.)	–	0 (ref. cat.)	–	0 (ref. cat.)	–	0 (ref. cat.)	–	0 (ref. cat.)	–	0 (ref. cat.)	–	0 (ref. cat.)	–
•Secondary school	5.29 (−39.8; 50.4)	0.8	−0.33 (−2.65; 2.00)	0.8	0.40 (−1.96; 2.77)	0.7	−0.50 (−1.40; 0.40)	0.2	**−2.62 (−4.30; −0.94)**	**0.002**	−0.57 (−2.16; 1.02)	0.4	−0.20 (−0.81; 0.41)	0.5
•High school	−17.6 (−64.2; 29.0)	0.5	−1.33 (−3.70; 1.03)	0.3	0.77 (−1.64; 3.17)	0.5	−0.72 (−1.63; 0.20)	0.12	**−2.43 (−4.13; −0.72)**	**0.006**	−0.43 (−2.01; 1.15)	0.6	−0.08 (−0.69; 0.53)	0.8
•University/higher	9.63 (−36.2; 55.5)	0.7	−0.01 (−2.33; 2.31)	0.9	0.41 (−1.96; 2.77)	0.7	−0.68 (−1.28; 0.22)	0.14	**−2.66 (−4.33; −0.99)**	**0.002**	−0.69 (−2.30; 0.92)	0.4	−0.08 (−0.69; 0.52)	0.8
Currently working Yes vs no	−0.66 (−37.8; 36.5)	0.9	−0.89 (−2.72; 0.94)	0.3	0.20 (−1.66; 2.07)	0.8	−0.08 (−0.79, 0.63)	0.8	−0.53 (−1.85; 0.80)	0.4	−0.32 (−1.50; 0.85)	0.6	−0.01 (−0.45; 0.43)	0.9
Presence of comorbidities Yes vs no	3.98 (−6.48; 14.5)	0.5	0.29 (−0.25; 0.82)	0.3	−0.02 (−0.57; 0.25)	0.9	0.04 (−0.17; 0.24)	0.7	0.33 (−0.05; 0.72)	0.09	0.10 (−0.19; 0.39)	0.5	−0.01 (−0.15; 0.13)	0.9
Trunk site vs others	5.76 (−21.5; 33.0)	0.7	0.99 (−0.34; 2.31)	0.14	0.02 (−1.32; 1.37)	0.9	0.05 (−0.46; 0.56)	0.9	−0.34 (−1.29; 0.62)	0.5	−0.53 (−1.17; 0.10)	0.09	−0.09 (−0.41; 0.23)	0.5
Presence of multiple lesions Yes vs no	−23.5 (−82.9; 36.0)	0.4	1.82 (−1.14; 4.77)	0.2	−0.24 (−3.26; 2.77)	0.9	0.80 (−1.10; 0.43)	0.17	1.41 (−0.72; 3.55)	0.2	−0.01 (−0.68; 1.14)	0.9	−0.21 (−0.93; 0.51)	0.6
Cancer stage at diagnosis:														
•In situ	0 (ref. cat.)	–	0 (ref. cat.)	–	0 (ref. cat.)	–	0 (ref. cat.)	–	0 (ref. cat.)	–	0 (ref. cat.)	–	0 (ref. cat.)	–
•IA-IB	12.2 (−23.3; 47.8)	0.5	0.34 (−1.51; 2.19)	0.7	0.44 (−1.44; 2.32)	0.6	−0.29 (−1.01; 0.43)	0.5	0.23 (−1.10; 1.57)	0.7	0.23 (−0.68; 1.14)	0.6	**0.52 (0.07; 0.97)**	**0.024**
•II-III-IV	**−44.8 (−87.2 ; −2.43)**	**0.038**	−1.68 (−3.85; 0.50)	0.13	0.09 (−2.13; 2.30)	0.9	−0.04 (−0.88; 0.81)	0.9	−0.39 (−1.96; 1.18)	0.6	0.52 (−0.59; 1.62)	0.4	−0.12 (−0.66; 0.42)	0.7
Time from surgical resection, 10-month increase	0.44 (−2.39; 3.28)	0.8	−0.10 (−0.23; 0.03)	0.14	−0.09 (−0.22; 0.05)	0.2	−0.00 (−0.05, 0.05)	0.9	0.02 (−0.07; 0.12)	0.6	0.02 (−0.04; 0.09)	0.5	−0.02 (−0.06; 0.01)	0.15

*PRISM* Pictorial Representation of Illness and Self Measure; *ACP* disease prognosis and acceptance; *CON* treatment concerns/future disease risk; *CARE* care delivery/communication; *SUP* supportive care; *SOC* social circumstances; *Coeff.* coefficient; *CI* confidence intervals; *ref. cat.* reference category

For PRISM, ACP, CARE, SUP and SOC subscales, a lower score indicates a lower quality of life; for CON subscale, a higher score indicates a lower quality of life

¶ Limbs, face, scalp, hands, feet, or genitals

Statistically significant results are reported in bold

As a separate, additional analysis, all models were re-run after excluding 5 high-leverage observations, with virtually identical results.

## Discussion

The main objective of this study was to investigate the measurement of global burden of suffering and quality of life impairment in patients with previous excision of skin MM, particularly assessing PRISM as a potentially useful tool in clinical practice. With reference to the main objective, it is possible to observe that in the investigated population, their perception of the burden associated with MM does not appear either particularly dramatic or disabling. This may be argued considering the results provided from both PRISM, which is a generic instrument, and MCQ-28, which specifically addresses several psycho-oncological domains deemed important to MM survivors (Table 2).

Even though it is hard to compare the PRISM scores recorded in our patients with those of other dermatological conditions published in the literature (due to a great heterogeneity of study settings, designs, populations and diseases themselves), some considerations may be made in this regard. Bearing in mind that lower scores of PRISM correspond to a higher level of suffering, the mean PRISM score in our population was quite similar to that found in 83 cancer survivors investigated in a previous study [22]. On the other hand, mean scores for patients affected with potentially less impacting diseases, including non-life-threatening conditions, such as liver transplant recipients and patients with liver cirrhosis [24], chronic urticaria [25], psoriasis [26], chronic inflammatory vulvar diseases [17], chronic cutaneous ulcers [27], ulcerative colitis [28] or tinnitus [29], were lower than that found in MM patients. This seems to indicate that all these diseases cause greater suffering than MM, an apparently paradoxical datum that could have several possible explanations. In our experience, albeit limited to subjects affected with inflammatory genital disorders, disease-related symptoms are the main determinants of the PRISM score [17, 18]. As previously excised, MM does not usually cause symptoms, unlike many of the other non-neoplastic diseases listed above; this could at least partially justify the difference in mean PRISM scores between conditions characterized by chronic, distressing symptoms and others without. Moreover, the vast majority of our patients were affected with low-stage MM, thus both with a normally good prognosis and not subjected either to close and invasive follow-up or systemic treatments. Furthermore, the time elapsed since MM excision was rather long, being about 36 months, with a quarter of patients who had undergone surgical resection over 72 months previously. Taken together, these aspects could have made the impact of MM less pressing on the lives and perceived well-being of patients.

However, it remains to be established whether the relatively high PRISM scores found in our study patients are due to its low propensity to intercept the emotional burden related to MM or to the characteristics of our population. A possible answer to this question is provided by the multiple regression models. In fact, MM stage was found to be significantly associated with PRISM scores. In particular, patients with stages II, III and IV provided significantly lower PRISM scores than subjects with MM in situ (Table 4). Therefore, the relatively low mean PRISM scores found in our study population may be consistently conditioned by the fact that about 80% were affected with superficial MM. Unlike the MM stage, time from surgical resection was not a determinant of the PRISM score.

The analysis of the correlation with MCQ-28, which is a questionnaire designed specifically to assess the health-related quality of life in MM survivors, provides interesting data on the reliability of PRISM to capture the degree of suffering from MM. Indeed, a significant correlation was found between PRISM and some MCQ-28 subscales, namely disease prognosis and acceptance (ACP), treatment concerns/future disease risk (CON) and social circumstances 2 (SOC2). This means that PRISM appears to be an effective indicator of patients’ acceptance of their condition as well as their propensity to look to the future with optimism, which are the issues addressed by ACP. PRISM also seems capable of measuring patients’ concerns about the risks of MM, both for themselves and for their relatives (CON items), and comfort to be intimate with their partners (SOC2 item). It is not surprising that the level of correlation between PRISM and these MCQ-28 items was weak (Table 3). In fact, PRISM and tools assessing the quality of life, like MCQ-28, quantify items that partly overlap each other, but are not exactly the same. PRISM aims to assess the extent of suffering associated with illness, which is not just the mere result of the illness itself, but is determined by the perception that a subject has of his/her own state. The tools that claim to assess health-related quality of life focus mainly on the direct effects of illness on different fields of the patient’s life. Consistent with this, it is probably not a coincidence that PRISM was correlated to aspects of quality of life more related to abstract feelings and emotional states, like those addressed by ACP and CON subscales, than to concrete and objective needs, such as those assessed by the care delivery/communication (CARE) and supportive (SUP) subscales.

With the exception of a few associations, mostly that between PRISM and MM stage, no other variables, either disease or patient-related, were found to significantly affect the scores of the two measuring tools. This finding was rather unexpected, since the detrimental effect of a disease on suffering and quality of life is usually mediated by personal factors, such as age, marital status and educational level. It is worthy of note that higher educational attainment was found to be inversely associated with the scores belonging to the SUP subscale. It can be assumed that subjects with a higher level of education, and perhaps higher awareness of their disease, require more support from health facilities and relatives.

Our study has some limitations. The PRISM tool depends on an interviewer and cannot be performed alone. This may inhibit patients in answering and may lead to partial mystifications of their real perception of the disease-related burden. Reliance on self-reported data is a somewhat unavoidable potential weakness of questionnaires, like MCQ-28. There is a strong numerical discrepancy in relation to the stage of MM, which however reflects the real life of our patients. Relevant comorbidities, potentially conditioning the patient’s perception of his/her health status, were not considered in detail but only as present or absent. A formal process of cross adaptation of the translated version of MCQ-28 was not carried out. A review of the Italian version of MCQ-28 by two independent experts led to a minimal culturally specific adaptation. Finally, given the limited number of patients undergoing a surgical resection within the previous 12 months, the data pertaining SURG subscales were too few to allow a meaningful multivariate analysis for these items.

In conclusion, based on our results, PRISM can be considered a valid, reliable and feasible system for quantifying some aspects of the quality of life in MM patients. PRISM seems particularly effective in capturing the domains correlated with the emotive dimension of MM, such as MM-related concerns and the willingness to face life. As expected, PRISM is affected by the stage of MM. The fact that we included mostly patients with earlier stages of MM probably resulted in an overall rather modest level of suffering and deterioration in the quality of life, as measured with PRISM and MCQ-28. Because of its prerogatives, PRISM seems particularly suitable in clinical practice, especially for intercepting unexpressed discomfort that requires adequate support. In particular, PRISM could help to identify subjects afflicted by worries that they are unable to communicate due to difficulties in verbalizing them or shame or lack of dialogue with the caregivers. These subjects may be advised to receive support from figures specialized in emotional support, as an integral part of the MM follow-up.

## Supplementary Information

High resolution image (TIF 63 kb)

ESM 2 (DOCX 21 kb)
