# Supplementary figures and images for: Assessment of the perceived burden associated with Malignant Melanoma with Pictorial Representation of Illness and Self Measure (PRISM) and Melanoma Concerns Questionnaire (MCQ-28)
Source: Support Care Cancer. 2022 Jan 15;30(4):3643–53. doi: 10.1007/s00520-021-06760-2 (PMC8857156; doi:10.1007/s00520-021-06760-2)

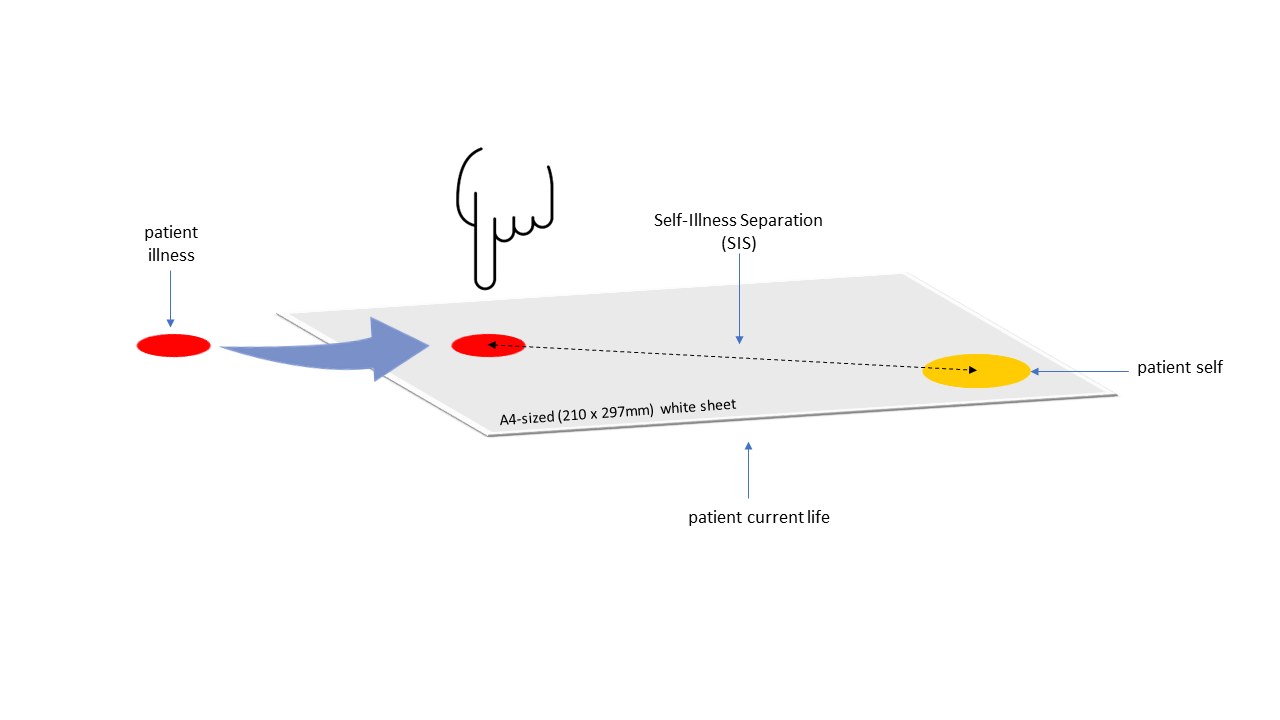

Supplement: Supplementary file 1 [file 520_2021_6760_Fig3_ESM.png]

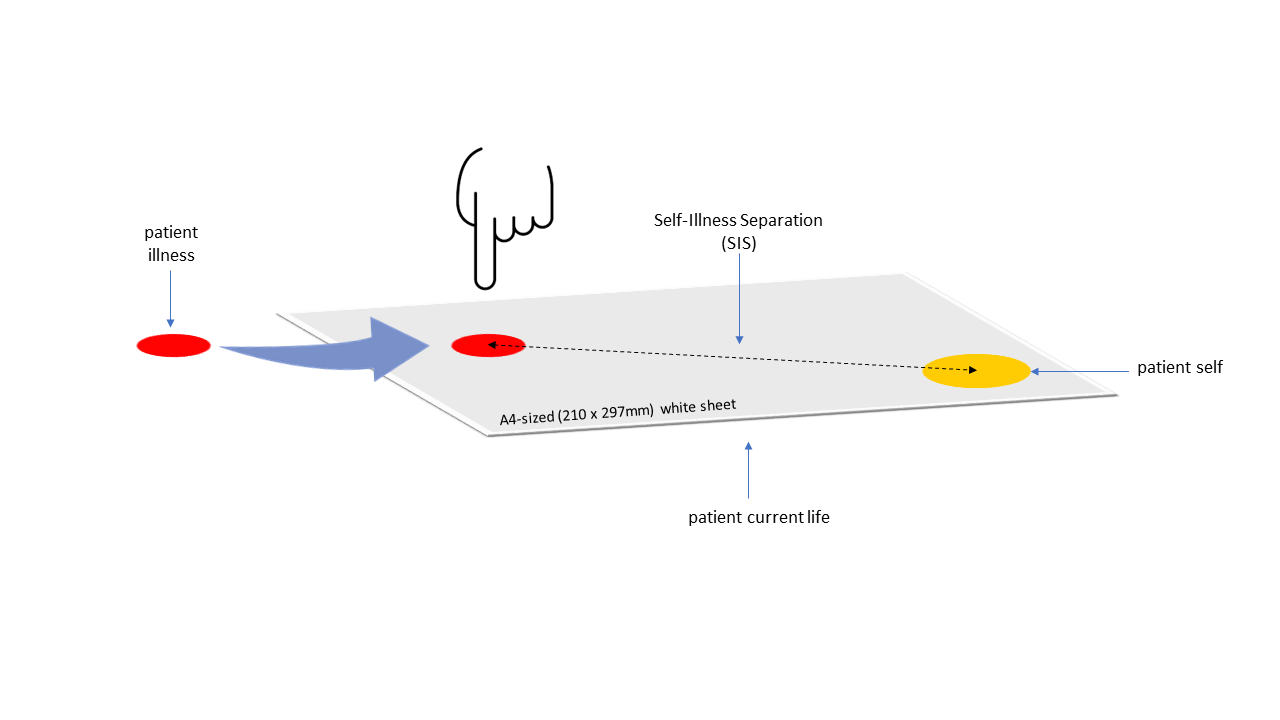

Supplement: Supplementary file 2 — High resolution image (TIF 63 kb) [file 520_2021_6760_MOESM1_ESM.tif]
