# Supplementary material for: Assessment of the perceived burden associated with Malignant Melanoma with Pictorial Representation of Illness and Self Measure (PRISM) and Melanoma Concerns Questionnaire (MCQ-28)
Source: Support Care Cancer. 2022 Jan 15;30(4):3643–53. doi: 10.1007/s00520-021-06760-2 (PMC8857156; doi:10.1007/s00520-021-06760-2)
Supplement: Supplementary file 3 — (DOCX 21 kb) [file 520_2021_6760_MOESM2_ESM.docx]

**Supplementary Table.** Melanoma Concerns Questionnaire© 28-item version (English and Italian versions)

|  |  |  |  | **Not at all**  **=**  **No** | **A little**  **=**  **Un po’** | **Quite a bit**  **=**  **Parecchio** | **Very much**  **=**  **Moltissimo** |
| --- | --- | --- | --- | --- | --- | --- | --- |
|  | **Have you had surgery for your melanoma in the last 12 months?  Yes □ No □  If yes, during the past 4 weeks** | **Ha subito un intervento chirurgico per il suo melanoma negli ultimi 12 mesi?  Si □ No □ Se si, durante le ultime 4 settimane** |  |  |  |  |  |
| 1 | Have you had swelling near your melanoma site? | Ha notato un gonfiore vicino al melanoma? |  |  |  |  |  |
| 2 | Have you had numbness at the site of your melanoma? | Ha avvertito intorpidimento vicino al melanoma? |  |  |  |  |  |
| 3 | Have you had problems with pain at or near your melanoma site? | Ha avuto dolore nella zona di insorgenza o vicino al melanoma? |  |  |  |  |  |
| 4 | How much have you worried about complications due to your melanoma surgery? | Quanto l’hanno preoccupata le complicazioni derivanti dall’intervento sul melanoma? |  |  |  |  |  |
| 5 | How much have you worried about the length of time needed for your melanoma surgery to heal? | Quanto l’hanno preoccupata i tempi necessari per la cicatrizzazione in seguito all’intervento sul melanoma? |  |  |  |  |  |
|  | **Since the diagnosis and treatment of your melanoma** | **A partire dalla diagnosi e dai trattamenti per il suo melanoma** |  |  |  |  |  |
| 6 | Have you worried about the increased risk of melanoma for other members of your family? | Quanto l’ha preoccupata l’aumentato rischio di contrarre il melanoma per gli altri membri della sua famiglia? |  |  |  |  |  |
| 7 | Have you had any regrets about your exposure to sunshine in the past? | Si è pentito/a di essersi esposto/a al sole in passato? |  |  |  |  |  |
| 8 | Have you felt concerned about conducting outdoor activities (e.g. working, swimming, walking, sport) which may result in exposure to the sun? | Le ha destato preoccupazione svolgere attività all’aperto (ad es., lavorare, nuotare, camminare, praticare sport) che potessero comportare un’esposizione al sole? |  |  |  |  |  |
| 9 | Have you considered making any significant changes to how you will live your life in the future? | Ha considerato di apportare dei cambiamenti importanti al modo in cui condurrà la sua vita in futuro? |  |  |  |  |  |
| 10 | Have you worried whilst waiting for results of medical tests? | Quanto si è preoccupato/a mentre attendeva i risultati delle sue analisi? |  |  |  |  |  |
| 11 | Have you felt confused by information about your diagnosis or treatment from different sources (e.g. internet)? | Le informazioni sulla diagnosi o sulla terapia provenienti da fonti diverse (ad es., internet) le hanno creato confusione? |  |  |  |  |  |
| 12 | Have you felt hopeful for the future? | Ha guardato al futuro con speranza? |  |  |  |  |  |
| 13 | Have you felt able to face the challenges ahead? | Si è sentito in grado di affrontare le difficoltà che si presenteranno sul suo cammino? |  |  |  |  |  |
| 14 | Have you felt able to cope with your diagnosis of melanoma? | Si è sentito in grado di affrontare la sua diagnosi di melanoma? |  |  |  |  |  |
| 15 | Have you felt able to deal with the shock of being diagnosed with melanoma? | Si è sentito in grado di affrontare lo shock dovuto alla diagnosi di melanoma? |  |  |  |  |  |
| 16 | Have you felt able to carry on with things as normal? | Si è sentito in grado di andare avanti con la sua vita quotidiana normalmente? |  |  |  |  |  |
| 17 | Have you felt able to feel positive? | Si è sentito in grado di avere un atteggiamento positivo? |  |  |  |  |  |
|  | **During the past 4 weeks** | **Durante le ultime 4 settimane** |  |  |  |  |  |
| 18 | If you work, have you had support from your employer to continue working during your treatment (e.g. flexible/reduced hours) | Se ha un lavoro, il suo datore le è venuto incontro per consentirle di continuare a lavorare durante la terapia? | not applicable  =  non applicabile |  |  |  |  |
| 19 | How much has your melanoma doctor supported you? | Si è sentito/a sostenuto/a dal medico che la assiste nella cura del melanoma? |  |  |  |  |  |
| 20 | How much have your other healthcare professionals (e.g. nurse) supported you? | Si è sentito/a sostenuto/a dagli altri operatori sanitari (ad esempio l’infermiere) che si occupano di lei? |  |  |  |  |  |
| 21 | How much have your family members supported you? | Si è sentito/a sostenuto/a dai famigliari? |  |  |  |  |  |
| 22 | How much has your primary care doctor supported you? | Si è sentito/a sostenuto/a dal medico di base? |  |  |  |  |  |
| 23 | Have you felt able to contact the melanoma clinical staff if you needed to? | Qualora ne avesse avuto bisogno, si è sentito/a in grado di poter contattare lo staff clinico impegnato nella cura del melanoma? |  |  |  |  |  |
| 24 | Have you felt confident that a psychological support service would be available if you needed it? | Ha fiducia nel fatto che, in caso di necessità, potrà avere a disposizione un servizio di sostegno psicologico? |  |  |  |  |  |
| 25 | Have you been given enough time to think about the treatment options available to you? | É soddisfatto del tempo che le è stato dato per pensare alle opzioni di trattamento a sua disposizione? |  |  |  |  |  |
| 26 | Have you felt comfortable being intimate with your  partner? | Si è sentito a suo agio nei momenti di intimità con il suo partner? | not applicable  =  non applicabile |  |  |  |  |
| 27 | How much have those important to you been included in discussions about your treatment options? | Quanto sono state coinvolte le persone per lei importanti nelle discussioni sulle opzioni di trattamento? |  |  |  |  |  |
| 28 | Have you felt confident that your healthcare team communicate with you in a professional manner? | Si fida del fatto che il gruppo di operatori sanitari che la assiste comunichi con lei in modo professionale? |  |  |  |  |  |
